# *Chlamydiae* and *Mycoplasma* infections in pulmonary MALT lymphoma

**DOI:** 10.1038/sj.bjc.6603981

**Published:** 2007-09-18

**Authors:** E Chanudet, P Adam, A G Nicholson, A C Wotherspoon, R Ranaldi, G Goteri, S A Pileri, H Ye, H K Müller-Hermelink, M-Q Du

**Affiliations:** 1 Division of Molecular Histopathology, Department of Pathology, University of Cambridge, Box 231, Level 3, Lab Block Addenbrooke's Hospital, Hills road, Cambridge CB2 0QQ, UK; 2 Institute of Pathology, University of Würzburg, Würzburg 97080, Germany; 3 Department of Histopathology, Royal Brompton Hospital, London SW3 6PN, UK; 4 Department of Histopathology, Royal Marsden Hospital, London SW3 6JJ, UK; 5 Anatomia Patologica, Università Politecnica delle Marche, Torrette di Ancona 60020, Italy; 6 Unità Operativa di Emolinfopatologia – Università degli Studi di Bologna, Bologna 40138, Italy

**Keywords:** pulmonary MALT lymphoma, *Chlamydia*, *Mycoplasma*

## Abstract

*Chlamydia pneumoniae*, *Chlamydia trachomatis* and *Chlamydia psittaci* were detected at low frequencies (<20%) among 69 pulmonary mucosa-associated lymphoid tissue (MALT) lymphomas, 30 other lymphoproliferative disorders (LPD) and 44 non-LPD. The incidence of individual *Chlamydiae* was generally higher in MALT lymphoma than non-LPD, although not reaching statistical significance. *Mycoplasma pneumoniae* DNA was not detected.

Mucosa-associated lymphoid tissue (MALT) lymphoma arises from the MALT acquired as a result of chronic inflammatory or autoimmune disorder (Isaacson and Du, 2004). The inflammatory process and the accompanying immunological responses provide a microenvironment essential for malignant transformation and for the subsequent expansion of the transformed clone. This is best exemplified in gastric MALT lymphoma, which is caused by chronic *Helicobacter pylori* infection and can be effectively treated by eradication of the organism with antibiotics (Wotherspoon *et al*, 1993). Growing evidence indicates that the development of non-gastric MALT lymphoma is also associated with infection by microbial pathogens. *Borrelia burgdorferi* and *Campylobacter jejuni* are associated with cutaneous marginal zone B-cell lymphoma and immunoproliferative small intestinal disease, respectively (Cerroni *et al*, 1997; Lecuit *et al*, 2004). Recent studies show that *Chlamydia psittaci* is variably associated with ocular adnexal MALT lymphoma in different geographical regions (Ferreri *et al*, 2004; Chanudet *et al*, 2006) and eradication of the organism results in complete regression of the lymphoma in some cases (Ferreri *et al*, 2005).

The aetiological factors underlying the development of pulmonary MALT lymphoma are unknown. Nonetheless, *Chlamydia* sp. and *Mycoplasma pneumoniae* can cause chronic pulmonary diseases such as chronic bronchitis and adult-onset asthma (Bjornsson *et al*, 1996; Daian *et al*, 2000), and chronic infection with *C. pneumoniae* or *M. pneumoniae* has been linked to an increased risk of lung cancer (Pehlivan *et al*, 2004; Littman *et al*, 2005). *C. pneumoniae* and *C. trachomatis* infections, particularly the former, are also associated with an increased risk of lymphoma although their specific association with pulmonary MALT lymphoma is unclear (Anttila *et al*, 1998). The present study was thus designed to examine the potential association of *Chlamydiae and Mycoplasma* infection with pulmonary MALT lymphoma.

## MATERIALS AND METHODS

Archival formalin-fixed paraffin-embedded lung biopsies (mainly parenchymal biopsies, occasionally biopsies of the bronchial mucosa) of 159 patients from 3 geographical areas, obtained between 1983 and 2006, were analysed. In all cases, the lung was the primary site of the disease. Local ethical guidelines were followed for the use of archival paraffin-embedded tissues for research and such use was approved by the local ethics committees of the author's institutions.

DNA was purified from whole tissue sections, and checked for integrity by multiplex PCR amplification of variously sized human gene fragments (100, 200, 300 and 400 bp) (Chanudet *et al*, 2006). Of the 159 cases, 143 had adequate materials as judged by quality control PCR and were thus suitable for PCR-based screening of microbial pathogens. These included 69 primary pulmonary MALT lymphomas, 30 pulmonary cases with other lymphoproliferative disorders (other-LPD), and 44 lung biopsies without any histological evidence of an LPD (non-LPD) (Table 1).

The detection of *C. psittaci*, *C. pneumoniae* and *C. trachomatis* was carried out separately using a previously described Touchdown PCR (Chanudet *et al*, 2006). *Mycoplasma pneumoniae* was detected by PCR of the *Mycoplasma*-associated P1 adhesion protein gene as described previously (Hardegger *et al*, 2000). Stringent laboratory procedures for PCR set-up and product analysis were carefully followed to avoid any potential cross-contamination. PCR products were analysed by electrophoresis on 10% polyacrylamide gels. Positive PCR products were purified and sequenced using an ABI 377 DNA sequencer (ABI PRISM Perkin Elmer) to confirm the specificity of PCR. In each case, three independent PCRs were carried out for each microbial species and only cases with positive PCR results in at least two of the three independent reactions were regarded as positive (Ferreri *et al*, 2004; Chanudet *et al*, 2006).

Differences in the prevalence of infectious agents among various lymphoma subtypes and controls were analysed using Fisher's exact test (‘stats Package’ in R version 2.1.1).

t(11;18)(q21;q21) and t(1;14)(p22;q32) were screened in 33 cases of MALT lymphoma by interphase fluorescence *in situ* hybridisation in previous investigations (Ye *et al*, 2003).

## RESULTS

**Figure 1:**
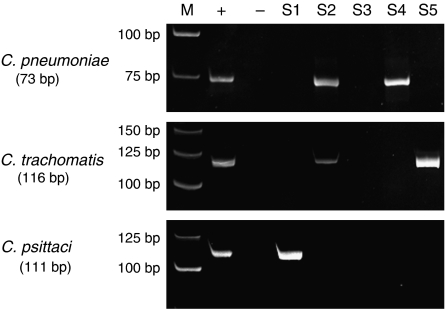
PCR detection of *Chlamydia pneumoniae*, *Chlamydia trachomatis* and *Chlamydia psittaci* DNA in pulmonary mucosa-associated lymphoid tissue (MALT) lymphoma specimen (10% polyacrylamide gel). M=molecular weight marker; +=positive control; −=negative control; S1–S5=different MALT lymphomas; S2=positive for both *C. pneumoniae* and *C. trachomatis*.

Of the 143 cases screened for the presence of *Chlamydia and Mycoplasma* (Figure 1), quality control PCR did not show any differences in the quality of DNA samples from MALT lymphoma or control groups, or from different geographical regions. Sequencing of PCR products confirmed specific amplification of all PCR primer sets.

Overall, 24 of 69 (35%) MALT lymphomas were positive for at least one of the three *Chlamydia* species, a frequency significantly higher than that in non-LPD (18%, *P*=0.043) but not other-LPD (27%, *P*=0.29). With the exception of two cases of MALT lymphoma (one from Italy and the other from UK), the presence of three *Chlamydia* species was mutually exclusive.

When the prevalence of individual *Chlamydia* species in various groups was examined within the same geographical region, no significant difference in the prevalence of each *Chlamydia* species was found between MALT lymphoma and other- or non-LPD group in all the three regions examined (Table 2). Nonetheless, the prevalence of *C. trachomatis* in MALT lymphoma (19%) was higher than those in other-LPD (5%) and non-LPD (4%) in Germany. *Chlamydia pneumoniae* was also detected more frequently in MALT lymphoma (23%) than other-LPD group (10%) in Italy. *Chlamydia psittaci* was found at low frequencies in MALT lymphoma, but absent in control groups in all three geographical regions. There was no correlation between *Chlamydia* positivity and age or sex of the patients. There was also no correlation between *Chlamydia* positivity and presence of t(11;18)(q21;q21) and t(1;14)(p22;q32) in 33 cases of MALT lymphoma from the United Kingdom and Germany, in which the translocation status was available from previous investigations. Remarkably, the four cases of DLBCL positive for a *Chlamydia* infection (three with *C. pneumoniae* and one with *C. trachomatis*) were all *de novo*. *Mycoplasma pneumoniae* was not detected in any MALT lymphoma and control cases despite the high sensitivity of the PCR, which was capable of detecting as few as three copies of *M. pneumoniae* genome.

## DISCUSSION

To our knowledge, this is the first study investigating the presence of infectious agents in pulmonary MALT lymphoma. Our results show low frequencies of *C. pneumoniae*, *C. trachomatis* and *C. psittaci* infection in pulmonary MALT lymphomas. The prevalence of the individual *Chlamydiae* was in general higher in pulmonary MALT lymphoma than non-LPD group. Nonetheless, this is not statistically significant; larger series of both MALT lymphomas and appropriate controls are required to assess the possible association of *Chlamydia* infection with pulmonary MALT lymphoma.

The low frequencies of *Chlamydiae* infection observed in pulmonary MALT lymphoma are analogous to the low prevalence of *B. burgdorferi* in cutaneous marginal zone B-cell lymphoma and *C. psittaci* in ocular adnexal MALT lymphoma (Cerroni *et al*, 1997; Chanudet *et al*, 2006). Together, these observations highlight an important issue for the study of microbial pathogens associated with non-gastric MALT lymphomas. The microbial pathogens associated with MALT lymphoma may reflect the spectrum of infectious agents that are capable of colonising and causing chronic infections at the respective mucosal sites. Unlike stomach where only *H. pylori* and its related species can survive the hostile acidic environment, lung is susceptible to a wide range of infectious agents. While gastric MALT lymphomas are invariably associated with *Helicobacter* species, pulmonary MALT lymphoma, similarly to other non-gastric MALT lymphomas, might be associated with several infectious agents. A recent study from Italy showed that 6 of the 13 *C. psittaci* negative ocular MALT lymphomas also responded to antibiotic treatment, suggesting the presence of other bacteria (Ferreri *et al*, 2006).

Remarkably, all cases tested in this study were negative for *M. pneumoniae* despite the high sensitivity of the PCR detection. This, together with the absence of *Chlamydia* infection in the majority of pulmonary MALT lymphomas, suggests the presence of other aetiological factors in the development of this lymphoma.

## Figures and Tables

**Table 1:** Demographic and histological characteristics of pulmonary MALT lymphomas and controls from different geographical areas

**Diagnosis**	**UK^a^**	**Germany^b^**	**Italy^c^**
*No. of patients*			
MALT L	35	21	13
Other-LPD	—	20	10
Non-LPD	20	24	—
			
*Median age (range)*			
MALT L	56 (37–78)	59 (35–78)	60 (35–84)
Other-LPD	—	59 (16–84)	53 (31–77)
Non-LPD	48 (14–66)	60 (24–75)	—
			
*Male/female ratio*			
MALT L	1.1	1.3	1.2
Other-LPD	—	1.2	0.4
Non-LPD	1	2.4	—

LPD=lymphoproliferative disorder; MALT L=mucosa-associated lymphoid tissue lymphoma.

a A total of 34 cases of MALT lymphoma from London and one case of MALT lymphoma from Cambridge. Control group includes 20 cases of non-lymphoproliferative disorders (mostly pneumonia and bronchiectasis) from London.

b All cases from Würzburg. Control groups consists of 20 cases of lymphoproliferative disorders other than MALT lymphoma (nine diffuse large B-cell lymphomas, three Hodgkin's lymphomas, two lymphomatoid granulomatosis, three mediastinal/anaplastic large cell lymphomas, one plasmocytoma, one mantle cell lymphoma and one NK-cell lymphoma) and 24 cases of non-lymphoproliferative disorders (mainly carcinomas).

c A total of nine cases of MALT lymphoma from Ancona and the remaining four cases from Bologna. Control group includes 10 cases of lymphoproliferative disorders other than MALT lymphoma (five diffuse large B-cell lymphomas, two follicular bronchiolitis, one mantle cell lymphoma, one Hodgkin's lymphoma and one lymphoplasmacytic lymphoma).

**Table 2:** Frequencies of *Chlamydiae* and *mycoplasma* detected in pulmonary lymphomas and controls from different geographical areas^a^

**Diagnosis**	**UK**		**Germany**		**Italy**		**Total**	
*Chlamydia pneumoniae*								
MALT L	2/35	6%	3/21	14%	3/13	23%	8/69	12%
Other-LPD	—	—	4/20	20%	1/10	10%	5/30	17%
Non-LPD	1/20	5%	2/24	8%	—	—	3/44	7%
								
*Chlamydia trachomatis*								
MALT L	8/35	23%	4/21	19%	2/13	15%	14/69	20%
Other-LPD	—	—	1/20	5%	2/10	20%	3/30	10%
Non-LPD	4/20	20%	1/24	4%	—	—	5/44	11%
								
*Chlamydia psittaci*								
MALT L	2/35	6%	1/21	5%	1/13	8%	4/69	6%
Other-LPD	—	—	0/20	0%	0/10	0%	0/30	0%
Non-LPD	0/20	0%	0/24	0%	—	—	0/44	0%
								
*Mycoplasma pneumoniae*								
MALT L	0/35	0%	0/21	0%	0/13	0%	0/69	0%
Other-LPD	—	—	0/20	0%	0/10	0%	0/30	0%
Non-LPD	0/20	0%	0/24	0%	—	—	0/44	0%

LPD=lymphoproliferative disorder; MALT L=mucosa-associated lymphoid tissue lymphoma.

a A case was regarded as positive if the screened bacterium was detected in at least two of three independent PCRs (Chanudet *et al*, 2006).
